# Physical Characteristics of the Leaves and Latex of Papaya Plants Infected with the *Papaya meleira* Virus

**DOI:** 10.3390/ijms17040574

**Published:** 2016-04-15

**Authors:** Anuar Magaña-Álvarez, Jean Carlos Vencioneck Dutra, Tarcio Carneiro, Daisy Pérez-Brito, Raúl Tapia-Tussell, Jose Aires Ventura, Inocencio Higuera-Ciapara, Patricia Machado Bueno Fernandes, Antonio Alberto Ribeiro Fernandes

**Affiliations:** 1 Núcleo de Biotecnologia, Universidade Federal do Espírito Santo, Av. Marechal Campos 1468, Vitória, Espírito Santo 29040-090, Brazil; anuar.magana@cicy.mx (A.M.-A.); jeanvencioneck@gmail.com (J.C.V.D.); tarcio_carneiro@hotmail.com (T.C.); ventura@incaper.es.gov.br (J.A.V.); patricia.fernandes@ufes.br (P.M.B.F.); alberto.fernandes@ufes.br (A.A.R.F.); 2 Laboratorio GeMBio, Centro de Investigación Científica de Yucatán A.C., Calle 43 # 130, Col. Chuburná de Hidalgo, Mérida, Yucatán 97200, Mexico; rtapia@cicy.mx; 3 Instituto Capixaba de Pesquisa, Assistência Técnica e Extensão Rural, R. Afonso Sarlo 160, Vitória, Espírito Santo 29052-010, Brazil; 4 Unidad de Tecnología de Alimentos, Centro de Investigación y Asistencia Tecnológica y Diseño del Estado de Jalisco A.C., Ave. Normalistas # 800, Col. Colinas de la Norma, Guadalajara, Jalisco 44270, Mexico; inohiguera@gmail.com

**Keywords:** papaya sticky disease, *Papaya meleira* virus, leaf midribs, laticifer, latex, pathogen-host interaction

## Abstract

Sticky disease, which is caused by *Papaya meleira* virus (PMeV), is a significant papaya disease in Brazil and Mexico, where it has caused severe economic losses, and it seems to have spread to Central and South America. Studies assessing the pathogen-host interaction at the nano-histological level are needed to better understand the mechanisms that underlie natural resistance. In this study, the topography and mechanical properties of the leaf midribs and latex of healthy and PMeV-infected papaya plants were observed by atomic force microscopy and scanning electron microscopy. Healthy plants displayed a smooth surface with practically no roughness of the leaf midribs and the latex and a higher adhesion force than infected plants. PMeV promotes changes in the leaf midribs and latex, making them more fragile and susceptible to breakage. These changes, which are associated with increased water uptake and internal pressure in laticifers, causes cell disruption that leads to spontaneous exudation of the latex and facilitates the spread of PMeV to other laticifers. These results provide new insights into the papaya-PMeV interaction that could be helpful for controlling papaya sticky disease.

## 1. Introduction

Papaya (*Carica papaya* L.) production currently amounts to approximately 12 million tons per year worldwide, and Brazil and Mexico are the main exporting countries [1]. Diseases are a growing problem in papaya cultivation and commercialization. Sticky disease, or meleira, which is caused by the *Papaya meleira* virus (PMeV), is responsible for severe economic losses that can affect between 50% and 80% of the total production [2].

Papaya tissues contain specialized cells known as laticifers, which are rich in proteases and alkaloids [3]. When infected by PMeV, papaya plants spontaneously exudate the latex from leaves and fruits. The latex oxidizes during atmospheric exposure, resulting in small necrotic lesions on the edges of young leaves and a sticky substance on the fruits that makes them unacceptable for consumption [4,5,6]. Light microscopy studies have been conducted to investigate the changes in papaya tissues infected with PMeV [7]. More recently, atomic force microscopy (AFM) was used to study the cell wall morphology [8,9] and the structural and mechanical properties of the cell walls of plants exposed to microorganisms [10,11].

Recently, reviewed review demonstrated the importance of highly sophisticated and innovative methods of analysis to improve our understanding of plant-pathogen interactions and increase crop yield [12]. For example, AFM could be used for screening disease-resistant breeding material. In the present study, we investigated the differences in the physical properties of the leaf midribs and latex of healthy and sticky diseased papaya plants using AFM and scanning electron microscopy (SEM).

## 2. Results

### 2.1. Topographical Analysis of the Leaf Midribs and Latex

**Figure 1:**
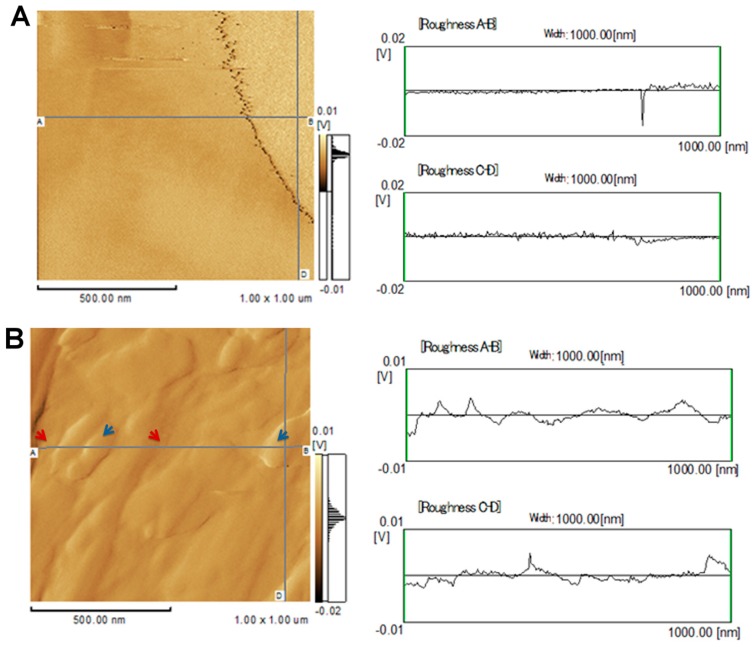

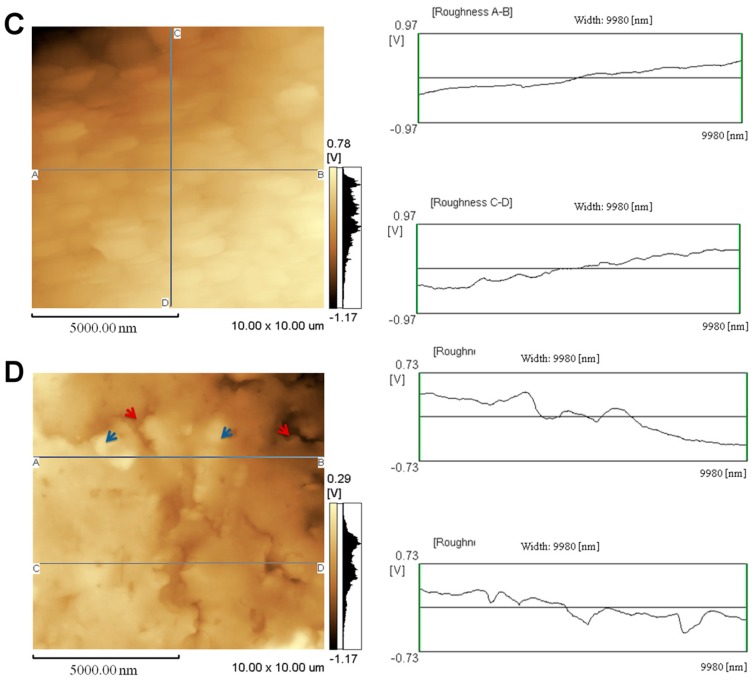
Representative two-dimensional atomic force microscopy (AFM) images and roughness profiles of the papaya leaf midribs (**A**,**B**) and latex (**C**,**D**) of healthy (**A**,**C**) and PMeV-infected papaya plants (**B**,**D**). Superficial differences between the samples from the healthy and infected plants. The infected samples exhibit valleys (red arrows) and peaks (blue arrows) that were not observed in the samples from the healthy plants.

Two-dimensional AFM images from different areas of the leaf midribs showed that the surfaces of the healthy plants are smoother than those of the sticky diseased plants (Figure 1A,B). The surfaces of both the healthy and diseased plants exhibited valleys, but these features were much deeper in the diseased plants (Figure 1B) than in the healthy plants (Figure 1A).

The two-dimensional AFM images obtained from latex were very distinct and revealed different surface features for healthy papaya fruits (Figure 1C) and those from PMeV-infected plants (Figure 1D). Indeed, the latex from healthy fruits had practically no roughness, and the surface was mostly flat.

**Figure 2:**
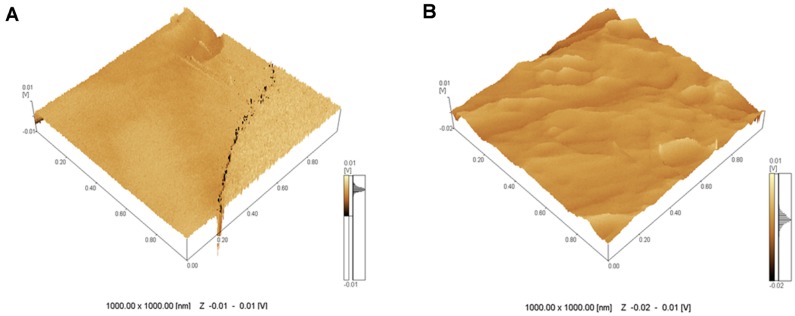

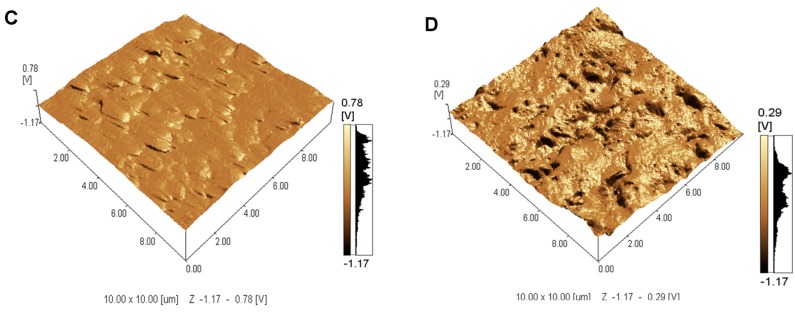
Representative three-dimensional AFM images of the leaf midribs (**A**,**B**) and latex (**C**,**D**) of healthy (**A**,**C**) and PMeV-infected papaya plants (**B**,**D**). The microstructures of the healthy leaf midribs and latex (**A**,**C**) show smooth surfaces, whereas the infected leaf midribs and latex have rough surfaces (**B**,**D**).

The three-dimensional images of the leaf midribs revealed a smooth microstructure with shallow valleys in the healthy plants and a rough microstructure (Figure 2A) with prominent ridges and deep valleys (Figure 2B) in the diseased plants. The images of the latex from the healthy papaya fruits included shallower valleys (Figure 2C) than those of the latex from the infected fruits (Figure 2D). Thus, the surface of PMeV-infected latex is more heterogeneous and has deeper valleys.

**Figure 3:**
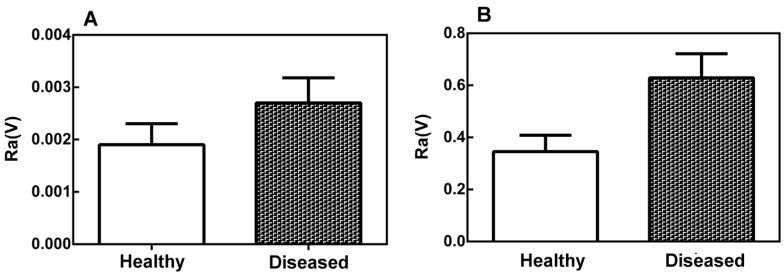
Structural roughness parameter Ra of the leaf midribs (**A**) and latex (**B**) of healthy and diseased papaya plants. The roughness (Ra) is the arithmetic mean of the deviations in the profile curve with respect to the midline of the basic length. The error bars represent the variations between biological replicates. The difference between the means is statistically significant (Mann–Whitney test, *p* < 0.05).

The values of the roughness parameter, Ra, (Figure 3) in the leaf midribs (A) and latex (B) were larger for the diseased plants than for the healthy plants, indicating that there is a significant difference (*p* < 0.05) between the surface roughness of the healthy and diseased plant tissues. This difference was more significant in the latex than in the leaf midribs.

### 2.2. Mechanical Properties of the Leaf Midribs and Latex

**Figure 4:**
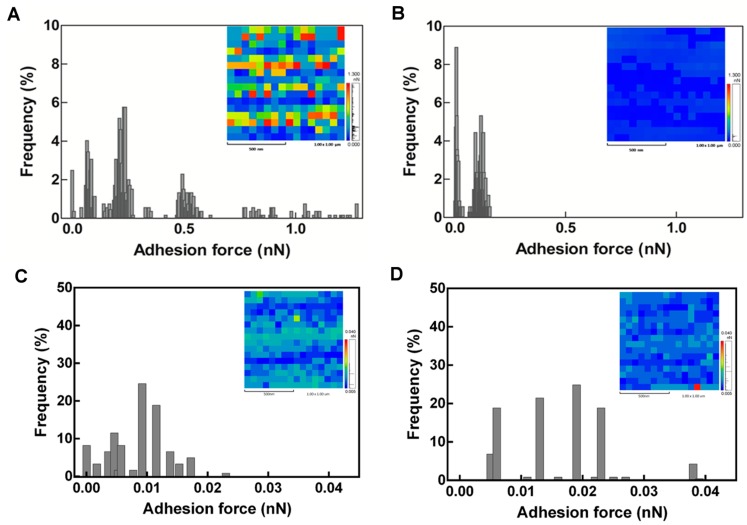
AFM maps and histograms of the adhesion force between the tip and the sample surface of the leaf midribs (**A**,**B**) and latex (**C**,**D**) of healthy (**A**,**C**) and diseased papaya plants (**B**,**D**). The vertical axis shows the frequency of the adhesion force in a 1000-nm lateral scan of 256 force curves, and the insets are the corresponding force maps.

The AFM adhesion force maps and histograms (Figure 4) provide information about the mechanical properties of the leaf midribs and latex of the healthy and diseased plants. The adhesion force maps are given in false white and black colors (Figure 4, inset). The frequency is presented as a percentage of the measured adhesion force in a 1000-nm lateral scan of 256 force curves.

According to the histograms obtained from an individual retraction curve, the diseased plants displayed increased heterogeneity of the surface midribs (Figure 4B) compared to the healthy plants (Figure 4A). Changes in the stiffness distribution are apparent in the color-coded adhesion force maps (Figure 4, insets). Soft areas (coded in blue) were predominant in the diseased plants (Figure 4B), whereas stiff domains (coded in red) were numerous in the healthy plants. This indicates that the cell walls of the healthy plants are more rigid than those of the diseased plants. The latex of the diseased fruits (Figure 4C) also displayed increased heterogeneity compared to the healthy plants (Figure 4D).

**Figure 5:**
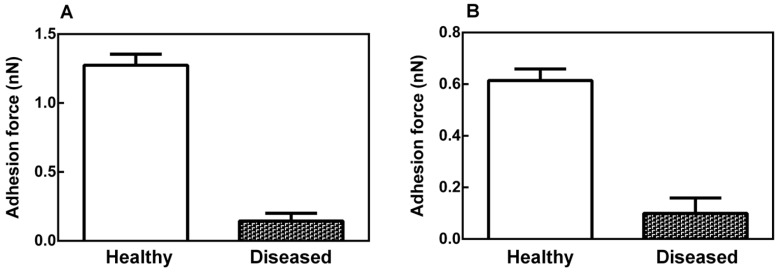
Average adhesion forces of the leaf midribs (**A**) and latex (**B**) of healthy and PMeV-infected papaya plants. The graphs show that the midribs and latex of the healthy plants have a greater adhesion force than those from the infected plants. The error bars represent the variations between biological replicates. The difference between means is statistically significant (Mann–Whitney test, *p* < 0.05).

The average adhesion force values of the healthy and diseased plants were 1.25 ± 0.05 and 0.15 ± 0.05, respectively (Figure 5).

### 2.3. Latex Examination by Scanning Electron Microscopy (SEM)

**Figure 6:**
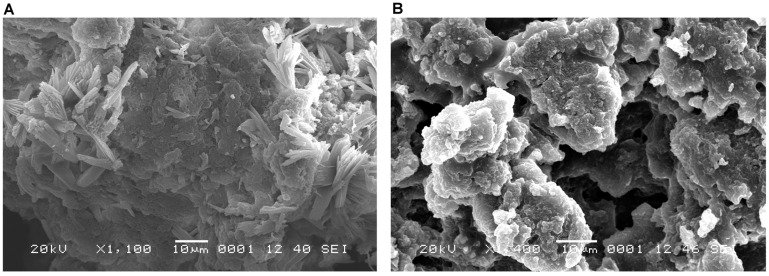
Scanning electron microscopy (SEM) images of the latex from healthy (**A**) and PMeV-infected papaya fruits (**B**). The samples of healthy plants’ latex shows more united and compact latex particles than those from infected plants, which appeared relatively sparse.

**Figure 7:**
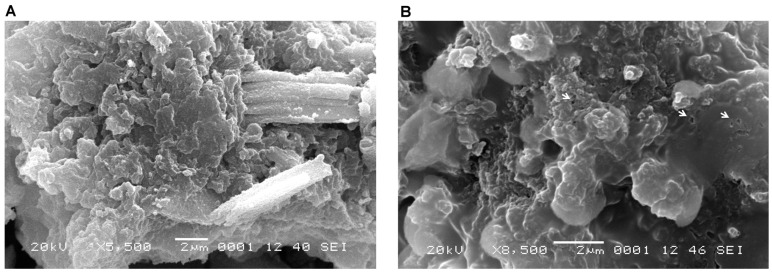
High-magnification SEM images of the latex of (**A**) healthy and (**B**) and PMeV-infected papaya fruits. In the infected latex small circles ranging from 40 to 50 nm, alterations in the structure of the latex and possible latex degradation are evident; these alterations were not observed in healthy latex.

An analysis of the SEM micrographs revealed that the latex particles in healthy papaya fruits (Figure 6A and Figure 7A) were closer together and more compact than those in fruits infected with PMeV (Figure 6B and Figure 7B). Small circles of approximately 40 nm, which likely arose because of the degradation of the latex by the viral infection, are observed in the diseased fruits (Figure 7B) but not in the healthy fruits (Figure 7A).

## 3. Discussion

The laticifers in *C. papaya* consist of thin-walled, greatly elongated, and highly branched ducts of anastomosed cells that are specialized for the production and storage of proteases and a secondary metabolite-rich fluid known as latex [13]. Laticifers are widely distributed in the aerial parts of the papaya plant and develop near the vascular bundles. The vascular bundles form the midribs and veins of the leaf [14]. One of the defense mechanisms of *C. papaya* plants is its latex, which is a hostile environment for pathogens; indeed, PMeV is the only pathogen that is confined to the lactiferous conducts of papaya latex and affects solidification in infected plants [4]. Therefore, it is important not only to study the latex alterations but also to investigate the vascular bundles where the laticifers are confined.

The material’s mechanical properties can be derived from the force *versus* displacement curves obtained using the AFM probe [15]. Physical structures or pressure differences between the surface tissues may originate from AFM tip indentations [16]. In the present study, the tip indentations causing pressure differences were avoided by using dried samples, as described by Aquije *et al.* (2010) [11], and the images and force data obtained from each sample in air were reproducible with repeated AFM operations.

The surfaces of the midribs and latex of the PMeV-infected plants showed regions with prominent ridges, deep valleys, and a rough microstructure (Figure 1 and Figure 2). Some components of the plant tissue act as first signals of infection and offer a certain level of protection against pathogens and abiotic stress [17,18,19]. However, PMeV can bypass the innate host defenses systems by altering the physical characteristics of the leaf midribs and latex of papaya plants.

In 2009, Rodrigues *et al.* [7] identified papaya laticifers as H_2_O_2_ producers. However, the levels of H_2_O_2_ production and accumulation were higher in the sticky diseased plants than in the healthy ones. This primary mechanism of strengthening the cell wall is common to plants that have been infected with a pathogen. However, based on our results, this mechanism did not seem to be effective in plants that were infected with PMeV for a long period (more than one year in our case), suggesting that PMeV likely has a specific mechanism of weakening the cell wall after the infection has been established.

We observed that virus activity leads to changes in the microstructures of the leaf midribs and latex of the infected plants, promoting increased surface roughness. This result corroborates the suggestion of Rodrigues *et al.* 2009 [7] that some classes of papaya cells, such as laticifers, behave differently when infected by the sticky disease virus.

Because the exudation of papaya latex requires tissue tapping under normal conditions, spontaneous latex exudation from sticky diseased papaya plants suggests that the plant laticifers burst [7]. Plants reduce the pore diameter of their plasmodesmata to limit the mobility of viruses [20], but PMeV counteracts this strategy by interfering with the physiology of the laticifers to compromise the assembly of the latex particles (Figure 6), thus increasing the water uptake [7] and making the latex from the sticky diseased papaya more fluid and translucent than its healthy counterpart [6].

PMeV is only found in laticifers, in close association with latex particles.It has been proposed that the swelling and subsequent rupture of laticifers in diseased papaya could be related to the virus’s strategy [7]. Because viral infection starts with contact between the virus and the cell membrane [21], the deep valleys may constitute weak points and areas that are susceptible to breakage, leading to microlesions used by the virus in the infectious process.

We observed that PMeV promotes changes in the leaf midribs, making them more fragile and susceptible to breakage (Figure 4 and Figure 5). We suppose that these morphological changes, which are associated with increased water uptake, increase the internal pressure of the laticifers promoting cell disruption and leading to spontaneous latex exudation and the spread of PMeV to other laticifers.

It is important to mention that the approximately 40-nm holes observed in the structure of the latex particles were only observed in the samples from the infected plants (Figure 6), as confirmed by SEM (Figure 7) and in a previous SEM study in Brazil, where holes were observed in the solid latex particles of PMeV-infected papaya plants. The authors found that the viral particles were confined to the latex and that the PMeV infection altered the lactiferous ducts, thereby preventing the aggregation of latex cells [7].

The presence of holes in the infected plants may result from latex degradation, which is likely caused by PMeV. The same type of degradation has been observed in rubber, in which the bacterium *Streptomyces* metabolizes the rubber latex biopolymer and triggers the degradation of isoprene, glycolipids, and lipopeptides, which are important compounds for rubber formation [22,23].

Knowledge of the physiological processes that underlie plant-pathogen interactions is crucial to improve crop performance. The results of this work provide new insights into the interaction between papaya and PMeV, which could contribute to the control of papaya sticky disease and the development of a genetically modified papaya.

## 4. Materials and Methods

### 4.1. Leaf Midrib and Latex Collection

*C. papaya* leaves of the same size and age were collected from healthy and diseased plants, and transverse leaf midribs sections were excised using a sterile razor blade. The sections were fixed with 0.1-M cacodylate buffer, pH 7.2, for 30 min; washed; dehydrated in a graded series of 30%, 50%, 70%, 90%, and 100% (*v*/*v*) ethanol; and critical point dried (CO_2_).

The latex was taken from green fruits of *C. papaya*, and the samples were collected in sterile 2-mL Eppendorf tubes using sterile toothpicks to pierce the surface of the green fruits displaying typical sticky disease symptoms that had a positive molecular PMeV diagnosis [24]. The latex from healthy fruits was used as a control.

### 4.2. Image Acquisition

Samples of the transverse leaf midrib sections were coded, and the AFM analyses were performed in a blinded manner. Five coded samples from each plant were attached to a glass slide using a small piece of double-sided adhesive tape, and five random points were examined; 25 images/points were assayed for each plant.

The AFM analysis of the latex samples was performed according to the procedure described by Aquije *et al.* (2010) [11]. Twenty microliters samples of healthy and PMeV-infected papaya latex were placed on microscope slide coverslips and allowed to dry at room temperature for 1 h. The latex samples were lyophilized prior to SEM.

All AFM images were captured via a Shimadzu AFM (SPM-9600 series, Shimadzu Corporation, Tokyo, Japan) using Si_3_N_4_ cantilever tips (model OMCL-TR, Olympus, Tokyo, Japan), with a nominal spring constant of 0.57 N/m and a resonance frequency of ≈73 kHz. The 512 pixels × 512 pixels AFM images were acquired with a scan rate of 1 Hz and a scan size of 1000 nm. Non-functionalized tips were used to measure the adhesion force. The force-distance measurements were obtained using dry samples and recorded in contact mode at room temperature (25 °C).

For SEM, the latex samples were mounted on aluminum stubs, sputter coated with 20-nm gold particles, and examined using a Shimadzu SSX 550 SEM (Shimadzu Corporation, Tokyo, Japan), operating at 12 kV. Three replicates were prepared from each of the lyophilized latex samples.

### 4.3. Statistical Analysis

The Mann–Whitney test was used to compare the healthy and diseased samples (*p* < 0.05).

## Abbreviations

PMeV	Papaya Meleira Virus
AFM	Atomic force microscopy
SEM	Scanning electron microscopy
H_2_O_2_	Hydrogen peroxide
